# Environmental degradation does not induce cortisol-measured stress in environmentally aware participants

**DOI:** 10.1371/journal.pone.0322464

**Published:** 2025-05-06

**Authors:** Rachelle K. Gould, Katrina Moreau, Brendan Fisher, David Gonzalez-Jimenez, Christine Vatovec

**Affiliations:** 1 Rubenstein School of Environment and Natural Resources, Gund Institute for the Environment, University of Vermont, Burlington, Vermont, United States of America; 2 Department of Biomedical and Health Sciences, College of Nursing and Health Sciences, University of Vermont, Burlington, Vermont, United States of America; 3 Centro de Formación y Desarrollo La Ceiba S.C., United States of America; 4 College of Nursing and Health Sciences, Osher Center for Integrative Health, Gund Institute for the Environment, University of Vermont, Burlington, Vermont, United States of America; Gujarat Institute of Desert Ecology, INDIA

## Abstract

It is well established that exposure to nature can reduce stress – but what if that “nature” is in a degraded state? We suggest a gap in research on nature—stress connections--and attempt to fill that gap. We conducted an experiment to test whether viewing photographs of polluted water would induce stress, as compared to photographs of clean water. In two conditions, we used sets of images that we digitally altered to be equivalent in every way except for the condition of the water. In the polluted-water treatment, all images depicted Harmful Algal Blooms (HABs), also sometimes called cyanobacteria blooms; in the clean-water (control) group, water looked free from HABs. Using a before-after-control-impact design, we tested pre- and post-intervention salivary cortisol to measure response to intervention exposure (i.e., photographs of lakes with or without HABs). We also collected qualitative data related to participants’ reflections on the images they observed, and quantitative data on their connectedness to nature and climate anxiety. Participants recognized the HABs and their negative effects. Yet our hypothesis—that participants who viewed HABs-infested images would have larger increases in cortisol—was not supported, even when considering participants with high and low measures of connectedness to nature and climate anxiety. We discuss possible explanations for the lack of effect found.

## Introduction

Stress is widely recognized as a powerful psychological state with diverse undesirable health consequences [1], and the interaction between stress and nature exposure is an important research area. Nature’s ability to ameliorate stress is well recognized [2]. Yet research on nature’s stress-reduction abilities often treats “nature” quite coarsely, with little detail. Current research on nature—stress links focuses on ecosystems that are perceived or presented (even if implicitly) as healthy, in good condition. Around the world, however, many ecosystems, including and perhaps especially many water bodies, are in degraded states [3]. How nature’s stress-reduction impacts may vary with ecosystem degradation is little studied.

**Fig 1:**
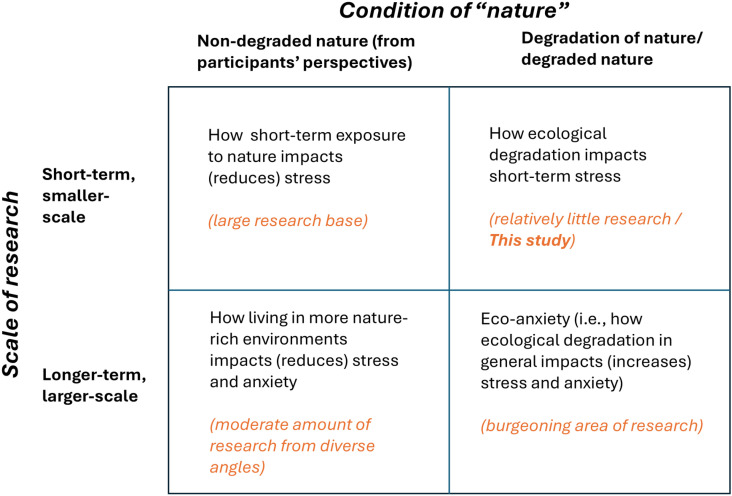
Schematic of the research landscape of interactions between stress and nature exposure. If the scale of the research (y-axis) and the condition of nature (x-axis) are considered, we suggest that this study addresses a gap in the current research landscape. The “non-degraded nature” column includes studies wherein the condition of “nature” is unspecified, and those in which participants perceive nature as non-degraded.

If “stress” is considered broadly, research on stress and nature has emerged in multiple disciplines and takes multiple forms. We suggest that this research landscape can be understood via two dimensions: the scale (both temporal and spatial) of the research, and the condition of the natural system involved – specifically, whether “nature” is understood as degraded or not (see Fig 1). Below, we summarize research in three of the four quadrants in Fig 1. We suggest that the current research landscape lacks exploration of the upper-right quadrant: of how small-scale nature degradation impacts stress. The present study works to populate that gap. We conclude the introduction with this study’s hypotheses, which directly address that research gap.

Extensive research suggests that nature exposure reduces stress (top-left corner of Fig 1), and salivary cortisol is among the primary biomarkers used to measure physiological stress response in research on nature exposure [4–6]. Early studies that examined cortisol levels in relation to environmental factors found that walking in and observing a natural area had a significant impact on salivary cortisol reduction as compared to time similarly spent in an urban setting [7,8]. Researchers have now conducted scores of similar studies, wherein they use salivary cortisol to measure stress response associated with nature exposure in diverse environments. Systematic reviews and meta-analyses of this work demonstrate that cortisol levels are consistently found to be significantly lower in natural environments as compared to urban settings [4,6,9]. Although most studies use direct nature exposure as treatment, some studies have explored the effects of videos on salivary cortisol [10].

Though most studies about stress-nature relationships are more acute (top-left corner of Fig 1), a smaller set of studies with diverse methods and disciplinary foundations explore stress-related impacts of chronic exposure to nature. These studies suggest that nature exposure can reduce chronic stress (bottom-left corner of Fig 1). Most of these studies do not specify the condition of the greenspace investigated; most use imprecise landcover classifications—e.g., greenery vs. not greenery. We provide three examples. One study measured cortisol in participants’ hair (which indicates about three months’ history of chronic stress); areas lower in “natural environment” (which included greenspace, woodland, agricultural land, and water as measured by satellite) were associated with higher chronic stress [11]. Another study found that the percentage of greenspace within residential areas correlated strongly with circadian cortisol patterns (with more greenspace associated with circadian patterns indicative of lower stress) [12]. A longitudinal study followed children for eight years (ages 3–9) and found that higher levels of greenspace (measured via satellite-assessed vegetation coverage) were associated with self-reported lower anxiety levels [13].

Research thus clearly demonstrates that nature exposure is associated with decreased stress. Yet at the same time, we know that witnessing environmental degradation can cause stress and associated anxiety (bottom-right corner of Fig 1). This field of study is coalescing under the term eco-anxiety, which is at present defined variously, but with most definitions related to “mental distress or anxiety associated with worsening environmental conditions” [14]. Recent work that aims to clarify definitions and streamline the field points out that eco-anxiety is a broad term that encompasses multiple emotional states, one of which is distress — our focus in this paper [15]. Though much research about eco-anxiety focuses on anxiety related to climate change generally [14,16], multiple strands of this work (especially research on how eco-anxiety interacts with ecological grief) focus on the distress associated with ecosystem degradation. These degradation-focused studies reveal substantial distress associated with degraded ecosystems [17,18].

Our review of the literature suggests a gap in this research landscape: The impacts of degradation have only been studied in more long-term, chronic situations – as eco-anxiety. We thus designed this study to explore the acute impacts of exposure to degraded nature on stress (upper-right corner of Fig 1). We measured stress via salivary cortisol. We conducted an experiment that compared changes in salivary cortisol before and after exposure to two conditions – one that highlighted water affected by Harmful Algal Blooms (HABs), and one that highlighted water without HABs. Our hypothesis is as follows:

H1: Cortisol levels will increase among study participants who receive the HABs treatment, as compared to the control (clean water) condition. Connectedness to nature and climate anxiety may interact with these effects.

## Methods

### Environmental degradation focus

The type of “degradation” upon which this study focuses is Harmful Algal Blooms (HABs). HABs occur when colonies of algae (small aquatic plants) experience population booms and produce substances that are harmful to people and other animals. The dominant causes of HABs are excessive nutrients (e.g., from agricultural-area runoff (from fertilizer use) or from sewage release into waters) and increased temperatures associated with climate change. Research demonstrates that HABs are highly noticeable and undesirable in many locations; they result in an array of socio-economic impacts [19], from decreases in shorefront property values [20] to harms to collective identity [21]. We conducted this study with HABs because they are a visible type of environmental degradation with high relevance for our study population.

### Participants and ethical approval

We visited three undergraduate courses, in six sessions, at the University of Vermont. Recruitment began Oct 1, 2021 and ended December 2, 2021. The classes we visited focused on: ecological economics (students mostly study environmental topics); human health and the environment (many students study nursing and medicine; some study environmental topics); and introduction to environmental studies (students mostly study environmental topics). We visited the full class for ecological economics and human health and environment classes; for introduction to environmental studies, we visited four sections (smaller meetings of roughly 15 students each).

This research was approved by the University of Vermont’s Institutional Review Board (STUDY00001734). The Institutional Review Board approved the following consent process. Roughly one week before sample collection in each course, students were provided a study information sheet electronically with a clear announcement explanation of its content (it was either emailed to students or posted on the course learning management system). On the day of the class visit, we reminded the class of the information sheet to which they had access, explained the study procedures, and alerted them that their participation in the study signaled their consent. We repeated that students had the option to decline participation and explained that not participating would not impact their class standing in any way. We did not collect identifying information, and the course instructor left the room prior to initiation of the experiment, so could have no knowledge of who participated and who did not. Very few participants declined to participate—roughly four students over the course of the study, less than one per class session. Those who chose not to participate were given an activity with a written reflection related to Lake Champlain.

### Study design

#### Baseline knowledge and awareness of HABs.

Amongst the U.S. population overall, awareness of HABs varies; a recent study, for example, found that 59% of U.S. adults surveyed were aware that HABs are a health threat [22]. For this experiment to be fully effective, participants must have at least baseline knowledge of HABs and their impacts—specifically, they must be able to visually identify HABs and know that they are undesirable and potentially dangerous (though HABs are visually unappealing in most cases, they can sometimes, to some viewers, appear beautiful or artistic). To increase the likelihood that all participants would enter the experiment with similar knowledge of HABs, roughly a month prior to the experiment, course instructors for the courses we visited shared with all participants a three-minute informational video, and made viewing it a course requirement. We chose to share the video a month prior to the experiment to ensure that all participants were exposed to the information, but to reduce any potential influence of priming. The video (viewable at https://youtu.be/nybrlK7iUjI)presented basic information about HABs—what causes them, and some of their most notable impacts on people and ecosystems.

### Conditions

**Fig 2:**
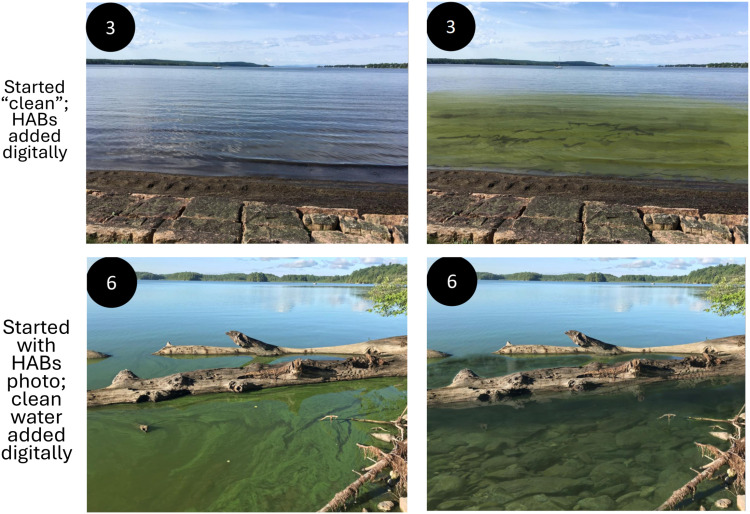
Examples of the image pairs used in our conditions.

This experiment has two conditions – a HABs treatment (85 participants) and a control (no HABs) condition (87 participants). To create the conditions, we selected ten photographs of water bodies from the eastern United States. We intentionally selected a diversity of photographs – e.g., those with different types of scenes, at different scales and from different perspectives. Half of these photographs were of HABs, and half of water unaffected by HABs. We then digitally modified all photos to create two sets of photographs that were identical in all ways except for the presence vs. absence of HABs. That is, we digitally removed the HABs from the images with HABs (and replaced them with HAB-free water), and digitally added HABs to the HAB-free images. Fig 2 demonstrates the resulting image pairs.

We created short, simple “workbooks” for each participant. Each workbook had either all HABs images, or all HABs-free images (half of each were digitally altered). The first seven pages of the workbooks contained photographs; the final four pages had a set of writing prompts and questions. Instructions asked participants to first spend a few minutes flipping slowly through the images, observing details of the scenes. Instructions then asked participants to write and draw responses to the prompts, which included items such as: “What would it feel like to swim in this water?”; and, after a prompt that the participant had accidently swallowed some of the water while swimming: “How do you feel about swallowing some of this water? What is going through your mind?” The primary goal of these prompts and questions was to provide participants with a meaningful reason to continue to look carefully, and repeatedly, at the images and think about the scenes they depicted – i.e., to engage cognitively with the experiment’s content.

### Connectedness to nature, climate anxiety, and demographics:

The final page of the workbook included a small number of quantitative items from established scales that measure connectedness to nature and climate anxiety, and record demographics. To measure connectedness to nature, we used seven items (with response options on a 1–7 Likert scale) [23]; we created a connectedness to nature index by taking the average score. To measure climate anxiety, we used a four-item scale that measured climate anxiety (with response options on a 1–7 Likert scale) [24]; we created a climate change anxiety index by taking the average score. We also asked two demographic questions (age and major).

### Sampling time

Most people experience a consistent daily pattern in cortisol levels: cortisol is highest in the morning, and then declines to reach a relatively steady state by early afternoon. For this reason, to increase the consistency of underlying cortisol levels in our sample, we selected only classes that met between 2pm and 5pm. The experiment took place as class began. Students settled into their seats and the instructors made announcements, then allowed the researchers to lead the class in the experiment.

The time it takes for cortisol to reach peak levels in saliva can vary depending on the type of stress, age, gender and links of the stressor to long-term memories [25–28]. Most studies suggest that peak post-stimulus salivary cortisol responses fall within a range of 25–30 min after stress cessation [27,29,30]. However, recent studies also differentiate between anticipatory cortisol stress response as opposed to reactive stress response. Reactive stress is the capacity or tendency to respond to an acute stressor (Scholtz, 2013), whereas anticipatory stress occurs when participants may already be aware of the nature of a given stressor; this means that anticipatory stress is often connected to previous knowledge of the stress factor [26]. Given that the stressor in our experiment was not an acute, dramatic event (e.g., a loud noise or a public performance, as in many other studies that induce stress), but instead depends crucially on prior knowledge of HABs and their impacts, the associated stress is likely more anticipatory than reactive.

Evidence suggests that cortisol stress response can be best perceived 10–16 minutes after the onset of anticipatory stress [26], and some research shows peak cortisol levels within the first 10 min post stress [28,31]. Thus, we allowed roughly 12 minutes to pass between the completion of pre-intervention saliva sample collection and the beginning of post-intervention sampling.

### Cortisol assessment

To collect and measure cortisol, we used Salimetrics® Cortisol Enzyme Immunoassay Kits. Participants collected saliva by passively pooling drool under the tongue and transferring it to a polypropylene cryovial via a SalivaBio Collection Aid. Samples (both pre- and post-intervention) were immediately stored in an ice-packed cooler and then transported within an hour of collection to storage -20°C until processing.

To prepare samples for analysis, we thawed, vortexed, and then centrifuged cryovials at 1500 x g for 15 minutes. All samples and reagents were brought to room temperature before being processed. Each plate contained six standards, high- and low-quality controls, zero, and non-specific binding wells in addition to participant samples. We ran all tests in duplicate and read them on a plate reader at 450nm with a secondary correction filter at 490nm.

We used the data reduction software MyAssays to interpolate the concentration of cortisol present for each sample. This software bases its interpolations on the optical density measurements using a four-parameter non-linear regression curve fit. We excluded samples with quantity insufficient to analyze, missing a pre or a post sample, and where condition categorization was unknown, which left 161 paired samples to analyze.

### Statistical analysis

We used SPSS 28 for statistical analysis. To account for individual variation in cortisol levels, our cortisol analyses centered on a calculated measure of proportional change in cortisol for each participant (we took the difference between the pre- and post- concentrations for each participant, then divided by the pre-intervention concentration).

We conducted multiple t-tests on these measures of proportional changes in cortisol concentration. We first compared results between the two conditions for the entire sample. We then wished to explore whether an effect might depend on participants’ levels of connectedness to nature or climate anxiety.

Even though we measured connectedness to nature and climate anxiety after the intervention, we used them as a way to split the sample, rather than as outcome variables. The main reason for this choice is the type of intervention we employed—short-term, laboratory-based, and not aimed at influencing connectedness to nature or climate anxiety. This intervention should not be expected to change these complex attitudes. We address past work that informs this expectation in the discussion.

We calculated the correlation between the connectedness-to-nature and climate anxiety scales; because the correlation was very low, we conducted separate analyses to explore whether treatment effects would be present only in people with high or low connectedness to nature, or with only high or low climate change anxiety. To divide the sample, we created four groups:

1. Low connectedness to nature: Only participants who scored one standard deviation or more below the mean for connectedness to nature;
2. High connectedness to nature: Only participants who scored one standard deviation or more above the mean for connectedness to nature;
3. Low climate anxiety: Only participants who scored one standard deviation or more below the mean for climate anxiety;
4. High climate anxiety: Only participants who scored one standard deviation or more above the mean for climate anxiety.

We then ran t-tests to compare percentage change between treatment and control participants for these four groups.

## Results

### Quality check

Our qualitative data allowed us to confirm that the vast majority of participants understood that the HABs treatment represented environmental degradation, and HABs specifically. Of the 85 participants who received the HABs treatment, all but two noted that the water was gross, disgusting, dirty, HABs-infested, or some variant of those terms. These two individuals said that they would swim in the water and otherwise answered in ways similar to participants in the clean (control) condition. These people were removed from the sample, as our research questions depend upon participant recognition that the water was not healthy. When emotions present in free responses are considered, participants in the HABs treatment almost all expressed disgust, concern/worry, and/or panic; participants in the clean condition almost all expressed relaxation, refreshment, and/or enjoyment.

### Hypothesis testing

Our hypotheses were not supported. Results suggest a null effect – i.e., condition did not impact cortisol levels. There is no significant difference between the percent change in cortisol for the two conditions (t = 1.452, df = 75.5). There is also no significant difference for any of the sub-groups investigated (Low CNS: t = -0.912, df = 16; High CNS: t = -0.665, df = 17; Low Climate Anxiety: t = -0.314, df = 17); High Climate Anxiety: t = -1.638, df = 31). See Table 2 for additional statistical results.

Table 1 reports the overall pattern of cortisol levels in our participants during the experiment; Table 2 reports the results of our analyses.

**Table 1:** Means (and standard deviations) for cortisol concentrations pre-and post-intervention, for both conditions.

	Pre-intervention cortisol	Post-intervention cortisol
HABs treatment	0.174 (0.119) µg/dL	0.156 (0.096) µg/dL
Control (no HABs) condition	0.156 (0.145) µg/dL	0.148 (0.110) µg/dL
Average	0.165 (0.132) µg/dL	0.152 (0.103) µg/dL

### Role of connectedness to nature and climate anxiety

We collected data on connectedness to nature and climate anxiety at the end of the experiment, so differences could have been impacted by the intervention. Scores for both indices, however, were statistically equivalent for participants in the two conditions (CNS: t = -0.391, 95% CI -0.310–0.208; CA: t = 0.189, 95% CI -0.213–0.258).

The connectedness-to-nature and climate anxiety scales are correlated, but extremely weakly (Pearson’s r = 0.226, 95% CI 0.052–0.386) (an r of 0.2 indicates a negligible correlation; the CI approaches but does not cross zero). Due to this low correlation (i.e., because the scales measure different phenomena), we ran additional analyses related to each scale. Specifically, we tested for treatment effects with both the high- and low-scoring participants on each of these scales, as described in the methods section (these analyses isolated participants below 1 SD and above 1 SD from the mean for each scale). Table 2 reports results from these analyses, none of which produced significant results.

**Table 2:** Analysis results. We conducted five analyses—the entire sample, then four split-sample groups that comprised only the low- and high-scoring participants for connectedness to nature and climate anxiety (i.e., participants who scored below one SD below the mean, or above one SD above the mean; see methods for details). For each analysis, the table shows: mean percent change in cortisol levels (pre-post intervention); standard deviations of those means; statistically estimated difference between the means for the two conditions; and lower and upper bounds of the 95% confidence interval of the difference between the means by condition). Differences between conditions were not significant for the entire sample).

Entire sample	Mean percent change in cortisol level	Standard deviation of mean percent change	Estimated difference between group means	CI lower bound (95% CI of the difference between group means)	CI upper bound (95% CI of the difference between group means)
HABs treatment	-0.052	0.254	−0.180	−0.43	0.06
Control (no HABs) condition	0.128	0.992			
**Split sample**					
Low CNS - HABs	0.010	0.331	−0.789	−02.8	1.2
- Clean	0.799	2.57			
High CNS - HABs	−0.040	0.282	−0.0922	−0.38	0.20
- Clean	0.522	.310			
Low CA - HABs	−0.034	0.322	−0.0405	−0.32	0.23
Clean	0.00680	0.237			
High CA - HABs	−0.108	0.180	−0.229	−0.52	0.063
Clean	0.121	0.546			

## Discussion

We found no difference in percentage change in cortisol between our two conditions, even when we analyzed sub-groups with high and low scores on connectedness to nature and climate anxiety. We also observed no difference in connectedness to nature or climate anxiety between conditions. In other words, looking at pictures of polluted water, even when participants recognize the water as a potentially toxic Harmful Algal Bloom, did not impact cortisol—even for sub-samples with high and low connectedness to nature and climate anxiety. Below we reflect on possible reasons for our null result, then we explain our decision to treat connectedness to nature and climate anxiety as moderator variables.

### Potential reasons for null results

One potential explanation for lack of observed change in cortisol is that the study population is already sensitized to environmental degradation. All participants are university students studying environmental issues, which suggests two intertwined potential explanations for the lack of observed effect. A first potential explanation is that all participants are already so highly aware of environmental degradation that looking at photographs of pollution for roughly ten minutes does not induce stress. A second potential explanation is that these participants, due to their high levels of environmental awareness, are chronically stressed by this knowledge and its implications [32]. It would be informative to try this study with a population that is both less familiar with environmental degradation and that spends less time exposed to reminders of that degradation.

Another potential explanation relates to the gap we identified in the introduction—short-term impacts of larger-scale environmental degradation—and how we might study that gap. Short-term impacts of environmental degradation may not induce the immediate “fight-or-flight” stress response with which cortisol is associated in the shorter term. We could look to evolution for this explanation; scholars suggest that one of the thorniest challenges of environmental action is that humans did not evolve to respond to the novel kinds of threats that environmental degradation poses [33,34] – we do not intuitively perceive many modern forms of environmental degradation as dangerous. Climate change is the most obvious example—there is nothing in our evolved set of danger responses that prepares us to react to elevated atmospheric CO2 levels (ibid.). Specific to this study, HABs are not instinctively dangerous – they are only perceived as dangerous if the viewer has knowledge about their negative consequences. It may, then, be difficult to detect the stress induced by HABs in the near term using physiological markers like cortisol. It is possible that this type of degradation may be associated only with lower-level, more chronic types of stress and anxiety (i.e., anticipatory stress [26]). These questions present fruitful areas for further research.

A third potential explanation for our null result is that photographs of environmental degradation are not impactful enough (or of the right kind of high-intensity impact) to precipitate detectable changes in cortisol. These images may, for instance, contribute to the more chronic stress described above (e.g., as measured in the hair of participants exposed to higher and lower levels of greenspace in their daily lives [11]). A different form of stimulus, a different way of presenting HABs to participants, might be more likely to induce a physiological stress response. For instance, it seems plausible that the physiological effect of short-term exposure to degradation would be stronger if the exposure is in person, in real life—or via media that more closely approximate real life, such as virtual reality or immersive video. Future research could explore cortisol-measured stress responses to environmental degradation as experienced in different ways, with the hypothesis that the closer the experience is to real life, the stronger the effect.

A final reason we may not have detected an effect may be a shortcoming in our research design. We waited roughly 12 minutes between the pre-intervention and post-intervention cortisol samples. This is at the low end of time in which intervention-induced changes in cortisol are observed. Few studies specifically explore how different waiting times may impact the effectiveness of salivary cortisol as a biomarker, with evidence suggesting that it may take anywhere from 15 minutes [35–37] to an hour [38] to observe cortisol changes after an intervention. There is not extensive research in this realm, but it seems that a longer waiting period than the one we employed may have allowed us to better detect changes in stress, as measured by salivary cortisol. Future research on the short-term stress impacts of environmental degradation would do well to repeat a study design such as that in Jimenez et al. [38], who sampled salivary cortisol after several time points following an intervention (15, 30, and 60 minutes).

### Connectedness to nature and climate anxiety

We treated high and low responses to connectedness to nature and climate anxiety scales as a way to split our sample (rather than as outcome variables). This is because past research suggested that our intervention was unlikely to produce a change in these phenomena. As expected, we did not observe any difference in these two scales between conditions.

Past research shows mixed results on the malleability of connectedness to nature by experimental intervention, with effects in the laboratory only seen with experiments explicitly designed to try to change connectedness to nature. Interventions that have led to observed changes include anthropomorphizing nature in a laboratory setting (vs. not anthropomorphizing nature) [39] and referring to nature as feminine (vs. masculine) [40]. One example of an intervention that did not lead to observed changes is watching a nature documentary (vs. a documentary not about nature) [41]—and there are likely many unpublished such studies, due to the File Drawer Problem—the tendency to “file away” and not publish null results [42]. The studies just listed are similar to ours in that they are short-term and laboratory-based. In contrast, complex, long-term engagements have ambiguous, complex impacts on connectedness to nature. As one example, learning about nature via a smartphone app (vs. not learning about nature) led to increases in connectedness to nature, but only indirectly via awe, and only for participants with higher levels of engagement [43]. As another example, a study of youth who participated in environmental education programs throughout the United States only weakly suggests programs’ influence on connectedness to nature (none of the programs studied exhibited change using the children’s version of the connectedness to nature instrument we used, but two of seven programs exhibited an increase with another instrument) [44]. This suite of ambiguous results, combined with the short duration of our intervention, undergird our decision to treat connectedness to nature as a relatively stable characteristic of respondents, rather than as a potential outcome variable.

We found no experimental research that explored how interventions impact climate anxiety. This may be due to the length of time the phenomenon has been studied: climate anxiety research (and particularly the scale we used, which was published in 2021) is much newer than connectedness-to-nature research (which began over two decades ago with the introduction of the now widely used scale [23]). Regardless, given the nature of the items in the climate anxiety scale (e.g., “I tend to worry when I hear about climate change, even with the effects of climate change may be some time away”), we did not expect our intervention to modify participants’ experience of this phenomenon.

### Suggestions for future research

This study suggests at least three potential arenas for future research: more exploration of the short-term effects of environmental degradation; study of these impacts on more diverse populations; and continued support for the publication of null-effect studies.

### Investigate short-term effects of environmental degradation

This study suggests that more experimental research to illuminate causation related to short-term effects of environmental degradation would be beneficial. This research falls into the top-right quadrant in Fig 1—the literature gap that we identified in the Introduction. Our null result populates this quadrant with more research, but it leaves many aspects of this gap unfilled. The dearth of literature in this quadrant makes it difficult to develop appropriate protocols to test these questions, and we hope our study can help others to refine study design – for instance, by inspiring the question of which biomarkers are most appropriate to measure physiological responses. One recent study, as an example, compares various biomarkers for stress and provides justification for salivary cortisol as appropriate in many contexts (including contexts like our study) [45]. Yet other studies suggest additional possibilities – for instance, the enzyme amylase rises more quickly than cortisol after stress-inducing events [46], and thus might be a more appropriate measure for immediate-term environmental stress. Knowing what analytical approaches yield insight, and which do not, can help build evidence to answer questions about short-term responses to degradation. These results, in turn, can inform further work that links short-term stress events to other outcomes of interest, such as environmental action.

### Conduct research with more diverse populations

There are myriad differences in salivary cortisol throughout the population, related to many factors [47,48]; this fact, combined with the results of our study, suggest that researchers should study the interface between stress and environmental degradation in diverse populations. More broadly than as relates only to cortisol or other biomarkers, biomedical research has for decades emphasized the importance of participant diversity—i.e., that participants of different backgrounds may have different psychological and biophysical reactions to treatments and interventions (both clinical and experimental) [49]. Psychological research similarly identifies the importance of participant diversity: teams of psychologists and anthropologists coined the apt acronym W.E.I.R.D. to refer to populations that are Western, Education, Industrialized, Rich, and Democratic – and therefore globally unusual [50,51]. This work points out that psychological tendencies that have been labeled as universal result from research with a small subset of the planet’s population. The role of participant diversity is garnering increasing attention in the study of the psychological impacts of nature exposure [52]. Though the focus in psychology is on the importance of studying non-WEIRD participants, the basic message is that we cannot assume that results in one population will mirror those in another.

To reflect on how our results might change depending on participant populations, we consider work on the importance of participant diversity in concert with past work on human-nature relationships and environmental justice. Extensive research on human-nature relationships makes clear that these relationships vary immensely across humankind – that diverse worldviews and knowledge systems mean that different populations consider their relationships with nature as quite different—and that relationships can change over time [53]. This type of difference might reasonably impact the relationship between nature degradation and stress responses. In addition, the expansive field of environmental justice research elucidates how history and a complex suite of practices have led to dramatic differences in the distribution of environmental benefits and burdens. Most relevant to the present study is that some communities, disproportionately communities of marginalized social groups, are disproportionately exposed to environmental burdens such as pollution [54,55].

Consideration of the research on participant diversity and environmental justice suggests that future research on the degradation-stress interface should likely focus on people experiencing disproportionate environmental harm. People at the frontlines and fencelines of experiencing environmental degradation are likely to have entirely different physical and emotional reactions to environmental degradation than our sample, which is largely sheltered from the daily impacts of such degradation. This emphasizes the importance of understanding how degradation may impact people’s daily lives. It is important to note that field work, including working with more difficult-to-find sample populations for experimental work, is expensive and difficult. It is difficult not only logistically (due, for instance to travel, finding and connecting with diverse communities) but also ethically. Ethical concerns are many; they include questions of how to work with impacted communities while dealing with sensitive issues of environmental injustice and how to respectfully engage with populations with different practices of communication and interaction—among myriad other issues. We suggest that researchers go the extra mile to conduct this work, and funders recognize that this important work will take more time and resource support.

### Publish null-effect studies

Our study also elucidates the importance of publishing studies with no significant results, wherein hypotheses were rejected. This practice has multiple clear benefits. One important benefit is the avoidance of the aforementioned “file drawer problem” – the tendency for null-result studies to be hidden in a researcher’s “file drawer,” instead of shared in such a way that others can learn from them [42]. This sharing can decrease the chance that researchers will replicate a study with a known effect, in these cases a null effect. It also helps share practices and lessons learned. Such learning can save researchers time and funders money, while simultaneously moving the field forward more rapidly.

A second benefit of publishing null results is that a norm of doing so, and ample avenues for doing so, will encourage researchers to take risks, which is the best way to make important leaps in understanding.

## Conclusion

In a controlled study of a salivary cortisol stress response to viewing photographs of clean vs. HABs-infested waters, we observed no effect of exposure to environmental degradation on short-term stress. We discuss three possible explanations for the lack of effect: our study population, which is highly sensitized to environmental degradation; human evolutionary history, which has not prepared us to perceive much environmental degradation as immediately dangerous; and the use of photographs, which may not be high-impact enough to induce a meaningful reaction in participants.

Understanding the links between stress response and pollution is becoming more relevant as pressures on the environment increase. We maintain that the research gap we identify in the introduction (of the impacts of environmental degradation on short-term stress) is worth exploring – particularly as it interacts with longer-term impacts on stress, as there is obviously no clear cut-off between “short-term” and “long-term” in this situation. Better understanding the links between degradation and stress will allow us to take the next step, with its crucial links to societal change: how does the combination of degradation and stress relate to societal action or inaction? This may help us avoid the potential paralysis induced by the overwhelming scope of environmental issues, and instead help us understand how to convert stress and emotional responses into productive action toward sustainability [17].

## Supporting Information

S1 File Supporting Information. (DOCX)

S2 Data GouldCortisolHABsData. (CSV)
