# Supplementary material for: Environmental degradation does not induce cortisol-measured stress in environmentally aware participants
Source: PLoS One. 2025 May 6;20(5):e0322464. doi: 10.1371/journal.pone.0322464 (PMC12054872; doi:10.1371/journal.pone.0322464)
Supplement: S1 File — (DOCX) [file pone.0322464.s001.docx]

**Supplementary Materials**

For the study: Gould et al., 2025, Environmental degradation does not induce cortisol-measured stress in environmentally aware participants.

**Climate Anxiety Scale used in this study**

*(With response options on a 1-7 Likert scale)*

*From reference [24].*

| I worry more about climate change than other people. |
| --- |
| Thoughts about climate change cause me to have worries about what the future may hold. |
| I tend to worry when I hear about climate change, even with the effects of climate change may be some time away. |
| I worry about how climate change may affect the people I care about. |

**Connectedness to Nature scale used in this study**

*(With response options on a 1-7 Likert scale)*

*From reference [23].*

| I often feel a sense of oneness with the natural world around me. |
| --- |
| I think of the natural world as a community to which I belong. |
| I recognize and appreciate the intelligence of other living organisms. |
| I often feel disconnected from nature. |
| When I think of my life, I imagine myself to be part of a larger cyclical process of living. |
| When I think of my place on Earth, I consider myself to be a top member of a hierarchy that exists in nature. |
| My personal welfare is independent of the welfare of the natural world. |

**Variable descriptions (see .csv file for complete dataset)**

NOTE: "Coded response" indicates not original data, but coding done by authors to aid in analysis.

| **Variable name** | **Description** |
| --- | --- |
| VialIDWorkbookNo | Unique subject identifier |
| DateSample | Date sample taken |
| Course | Course in which participant was enrolled |
| TreatmentCategory | Treatment category |
| PREAVGConc_VialA | Cortisol concentration pre-treatment |
| POSTAVGConc_VialB | Cortisol concentration post-treatment |
| Qual-CanyouseeyourselfswimminginthewaterintheimagesWhy | Qualitative Data: Answering question in variable name |
| CODED--SwimYESorNo | Coded response to Qual-CanyouseeyourselfswimminginthewaterintheimagesWhy (1=yes would swim; 0=no would not swim) |
| Qual-Whatwoulditfeelliketoswiminthiswater | Qualitative Data: Answering question in variable name |
| Qual-Canyouseeyourselffishinginthewaterintheimages | Qualitative Data: Answering question in variable name |
| Qual-why(toFishingQuestion) | Qualitative Data: Answering question in variable name |
| CODED--FishingYES | Coded response to Qual-why(toFishingQuestion); 1=yes |
| CODED--FishingYesbuthesitant | Coded response to Qual-why(toFishingQuestion); 1=yes, but with hesitation |
| CODED--FishingNoH20 | Coded response to Qual-why(toFishingQuestion); 1=no, because of the water quality |
| CODED--FishingNoOther | Coded response to Qual-why(toFishingQuestion); 1=No, for a reason other than the water |
| Qual-Pleasewriteaveryshort3-5sentencesstoryaboutonethingthathappenedon | Qualitative Data: Answering question in variable name |
| CODED--EDisgust | Coded response to Qual-Pleasewriteaveryshort…; 1=response indicated emotion of disgust |
| CODED--EFear | Coded response to Qual-Pleasewriteaveryshort…; 1=response indicated emotion of fear |
| CODED--EConcernworry | Coded response to Qual-Pleasewriteaveryshort…; 1=response indicated emotion of concern or worry |
| CODED--EPanic | Coded response to Qual-Pleasewriteaveryshort…; 1=response indicated emotion of panic |
| CODED--ESad | Coded response to Qual-Pleasewriteaveryshort…; 1=response indicated emotion of sadness |
| CODED--EDisappointment | Coded response to Qual-Pleasewriteaveryshort…; 1=response indicated emotion of disappointment |
| CODED--EAnger | Coded response to Qual-Pleasewriteaveryshort…; 1=response indicated emotion of anger |
| CODED--ENeutral | Coded response to Qual-Pleasewriteaveryshort…; 1=response indicated neutral or no emotion |
| CODED--EHappy | Coded response to Qual-Pleasewriteaveryshort…; 1=response indicated emotion of happiness |
| CODED--ERelaxed | Coded response to Qual-Pleasewriteaveryshort…; 1=response indicated emotion of relaxation |
| CODED--ERefreshing | Coded response to Qual-Pleasewriteaveryshort…; 1=response indicated emotion of refreshment |
| CODED--EFunorenjoyment | Coded response to Qual-Pleasewriteaveryshort…; 1=response indicated emotion of fun or enjoyment |
| CODED--EOthertypein | Coded response to Qual-Pleasewriteaveryshort…; emotions not coded in previous variables, typed in |
| Qual-HowdoyoufeelaboutswallowingsomeofthiswaterWhatisgoingthroughyourmind? | Qualitative Data: Answering question in variable name |
| Qual-Whatisyouremotionalreaction | Qualitative Data: Answering question in variable name |
| CODED--SwallowDisgust | Coded response to Qual-Whatisyouremotionalreaction…; 1=response indicated emotion of disgust |
| CODED--SwallowFear | Coded response to Qual-Whatisyouremotionalreaction…; 1=response indicated emotion of fear |
| CODED--SwallowConcernworry | Coded response to Qual-Whatisyouremotionalreaction…; 1=response indicated emotion of concern or worry |
| CODED--SwallowPanic | Coded response to Qual-Whatisyouremotionalreaction…; 1=response indicated emotion of panic |
| CODED--SwallowSad | Coded response to Qual-Whatisyouremotionalreaction…; 1=response indicated emotion of sadness |
| CODED--SwallowDisappointment | Coded response to Qual-Whatisyouremotionalreaction…; 1=response indicated emotion of disappointment |
| CODED--SwallowAnger | Coded response to Qual-Whatisyouremotionalreaction…; 1=response indicated emotion of anger |
| CODED--SwallowNeutral | Coded response to Qual-Whatisyouremotionalreaction…; 1=response indicated neutral emotion |
| CODED--SwallowHappy | Coded response to Qual-Whatisyouremotionalreaction…; 1=response indicated emotion of happiness |
| CODED--SwallowRelaxed | Coded response to Qual-Whatisyouremotionalreaction…; 1=response indicated emotion of relaxation |
| CODED--SwallowRefreshing | Coded response to Qual-Whatisyouremotionalreaction…; 1=response indicated emotion of refreshment |
| CODED--SwallowOther | Coded response to Qual-Whatisyouremotionalreaction…; emotions not coded in previous variables, typed in |
| CNS--Ioftenfeelasenseofonenesswiththenaturalworldaroundme | Item in the Connectedness to Nature Scale |
| CNS--IthinkofthenaturalworldasacommunitytowhichIbelong | Item in the Connectedness to Nature Scale |
| CNS--Irecognizeandappreciatetheintelligenceofotherlivingorganisms | Item in the Connectedness to Nature Scale |
| CNS--NEEDS.REVERSE.CODEIoftenfeeldisconnectedfromnature | Item in the Connectedness to Nature Scale |
| CNS--WhenIthinkofmylifeIimaginemyselftobepartofalargercyclicalprocess | Item in the Connectedness to Nature Scale |
| CNS--NEEDS.REVERSE.CODEwhenIthinkofmyplaceonEarthIconsidermyselftobe | Item in the Connectedness to Nature Scale |
| CNS--NEEDS.REVERSE.CODEMypersonalwelfareisindependentofthewelfareofth | Item in the Connectedness to Nature Scale |
| CCAnxietyWorryMoreThOthers | Item in the Climate Anxiety Scale |
| CCAnxietyWorryFuture | Item in the Climate Anxiety Scale |
| CCAnxietyWorryEvenThoughFarOff | Item in the Climate Anxiety Scale |
| CCAnxietyWorryPeopleCareAbout | Item in the Climate Anxiety Scale |
| Age | Age in years |
| Major | Major in collect |
